# Improving student engagement with a flipped classroom instruction model in Ethiopian higher education institutions: The case of Mattu University

**DOI:** 10.1371/journal.pone.0307382

**Published:** 2024-10-02

**Authors:** Mekonnen Haile Faro, Tariku Sime Gutu, Adula Bekele Hunde

**Affiliations:** 1 Department of Educational Technology and Information Management, Mattu University, Metu, Ethiopia; 2 Departments of Teacher Education and Curriculum Studies, Jimma University, Jimma, Ethiopia; Far Eastern University - Manila, PHILIPPINES

## Abstract

Currently, the flipped classroom instruction model has received greater attention for its multifaceted advantages in improving student learning by boosting learning environments. To this end, a flipped classroom model was employed in the current study with the purpose of increasing student engagement across all three dimensions—behavioral, emotional, and cognitive. To look at the improvement observed as a result of this model, this study used a mixed-methods design, mainly a convergent parallel design. In the present study, fifty-five students from two distinct academic departments participated. In addition, the instructors of the courses that were chosen to be taught using a flipped classroom model were also participants in this study. The study employed focus groups, interviews, self-report surveys, and classroom observation strategies to gather the necessary data. The quantitative data were analyzed using descriptive aggregate means and paired sampling t-tests, while the qualitative data were analyzed using a thematic data analysis approach. The findings revealed that a flipped classroom greatly improved each of the three dimensions of student engagement. Moreover, the findings showed that the instruction model improved student engagement by creating learning environments where students could participate actively in class activities, interact more effectively with teachers and other students as well as with learning resources, and acquire the necessary knowledge from the courses. Future research may employ a large sample size with a sufficient time frame to make the findings more applicable to different educational institutions.

## Introduction

Higher education institutions are required to play a significant role in a nation’s growth by producing competent and qualified professionals [1]. In order to fulfil this responsibility, educational institutions must guarantee that students receive a quality education that helps them make a substantial contribution to the social, political, and economic advancement of society [2]. In this regard, the process of instruction—specifically, the teacher-pedagogical approach—is crucial to ensuring high-quality learning for students [2]. According to studies, a student-centred approach plays a critical role in enhancing the quality of learning for students with active engagement [3]. Constructivist learning theory promotes this instructional approach as a helpful strategy for achieving student success and student engagement [4]. The constructivists highlight that the student-centred approach is an effective strategy to help students create their own knowledge by blending new experiences with what they already know and attributing high-quality learning to student engagement [5–7]. Active learning, constructivist pedagogy, fosters collaboration, innovation, communication, student-teacher accountability, in-depth learning, critical thinking, problem-solving, and reflective thinking, improving learning quality [12,13].This implies that student engagement is an important instructional aspect that requires attention from academics, as many have emphasized [8].

The term "student engagement" is a multifaceted concept lacking a precise definition. The term may stand for students’ sense of belonging, autonomy, involvement, interest, enjoyment, perceived value, persistent efforts, and the like [9,10]. Specifically, many academics accept the definition given by Fredericks and his colleagues, viewing it as comprehensive [11,12]. The definition given by these scholars conceptualizes this term as having three inherent dimensions, including behavioral, emotional, and cognitive [8]. The behavioral dimension refers to attending class frequently, completing assignments, participating in class activities, getting high grades, and so on. The second aspect, emotional engagement, denotes the student’s emotional response to academic subjects, peers, and teachers, promoting interaction and study dedication, influenced by their views, interests, and values. Cognitive engagement, which represents learning indicators such as diligent study, effectively finishing tasks, practicing self-control, keeping attention, and applying metacognitive methods, shows how well students can understand courses [8].

Astin’s theory of student involvement in college has a significant place in the history of student engagement. Engaged students are those who put in both mental and physical effort in their coursework, extracurricular activities, and interactions with teachers. The importance of student motivation and behavior in fostering improved learning and personal growth is highlighted by this student involvement theory [13]. Another theory that dealt with student engagement was self-determination theory (SDT). It is a motivational theory that outlines five mini-theories that illustrate how classroom dynamics can influence students’ motivation and engagement [14].

In addition, Barkley, who focused on student engagement at the class level, proposed that student engagement is the synergistic relationship between motivation and active learning [15].

Moreover, several studies have shown that, in keeping with Barkley’s approach, active learning strategies, including cooperative learning, small-group discussions, and problem-solving, can increase student motivation and engagement in the classroom [16]. In the digital era, student-centered instruction is being promoted over traditional methods, with innovative pedagogies like flipped classrooms enhancing active student engagement [17]. A flipped classroom is a teaching model that enhances student-quality learning, particularly in higher education, addresses pedagogical issues, and enhances student engagement and learning outcomes [18]. Moreover, flipped learning makes pupils more adaptive learners by combining various learning modes [19]. As per Jiang and Kamel Shaker Al-Shaibani (2022), adaptability is a valuable behavior that properly predicts student engagement [20]. Therefore, the current study employed the flipped classroom instructional model with the purpose of improving student engagement across behavioral, emotional, and cognitive domains.

### Rationale of the study

Ethiopia’s public universities have experienced growth and increased enrollment due to increased interest in higher education’s role in economic growth and the need for globally competent graduates [21]. The education system has expanded, introducing modular teaching, continuous assessment, and student-centered strategies, but these efforts have not improved teaching and learning processes, leading to student academic cheating and inflated grades [22]. On the other hand, studies revealed that teachers’ instructional approach was suppressive for student engagement in Ethiopian higher education, as most teachers adhered to a lecture approach only [23]. The present study aimed to increase student engagement as its primary goal. To this end, the study adapted the flipped classroom model to explore the impact of the model on student engagement at Mattu University, one of the public universities in the country. The model was adapted considering the model’s potential to address traditional teacher-centred challenges in higher education [24]. Currently, flipped classroom model that embraces digital technology is bringing about new paradigms in the instructional process in higher education, as indicated in various studies [25]. Studies revealed that flipped classrooms could enhance student engagement by creating conducive learning environments and allowing teachers to adapt their roles to students’ digital reliance [26]. Thus, the model was adapted, assuming that it could alleviate the prevailing problems related to teachers’ instructional approaches.

### Conceptual framework of the study

The flipped classroom model is rooted in socio-constructivist theory. The main emphasis of this theory is active learning and knowledge construction by students through social context and group work with teacher support [4]. The theory proposes that students build their own knowledge by combining their prior information with fresh experiences [5]. Similarly, the flipped classroom is a pedagogical strategy that facilitates student-centred or active learning. The model entails direct instruction shifting from individual to group settings, resulting in a dynamic and interactive atmosphere [27,28]. This model encourages students to actively construct meaning and control the learning process, combining traditional lecture-based methods with online video-based lectures and in-class problem-solving, promoting active exploration of the subject [29]. In other words, the model promotes student engagement in academic activities by rotating students between outside and inside classroom learning [30].

Different sources have proposed different strategies for implementing the flipped classroom model. For example, Dunn used procedures that held six points—planning, preparing learning materials, sharing, shifting, grouping, and regrouping—to put this strategy into practice [31]. Kim et al. (2014) proposed nine strategies that facilitate the learning environment in flipped classrooms. These included giving students their initial exposure prior to class, offering incentives for preparation, having mechanisms for assessment, making links between in-class and out-of-class activities clearly, offering well-structured guidance, giving assignments enough time, providing facilitation for building a learning community, offering adaptive feedback, and providing familiar and easily accessible technologies [32]. Roehling reduced those nine steps to just eight. These consisted of providing students with opportunities to learn outside the classroom, holding them accountable for pre-class preparation, assessing both pre-class and in-class activities, providing structured guidance, creating connections between in-class activities, pre-class materials, and learning objectives, allowing enough time to complete the in-class assignments, maximizing opportunities for faculty to interact with students, and offering free passes [33].

**Fig 1:**
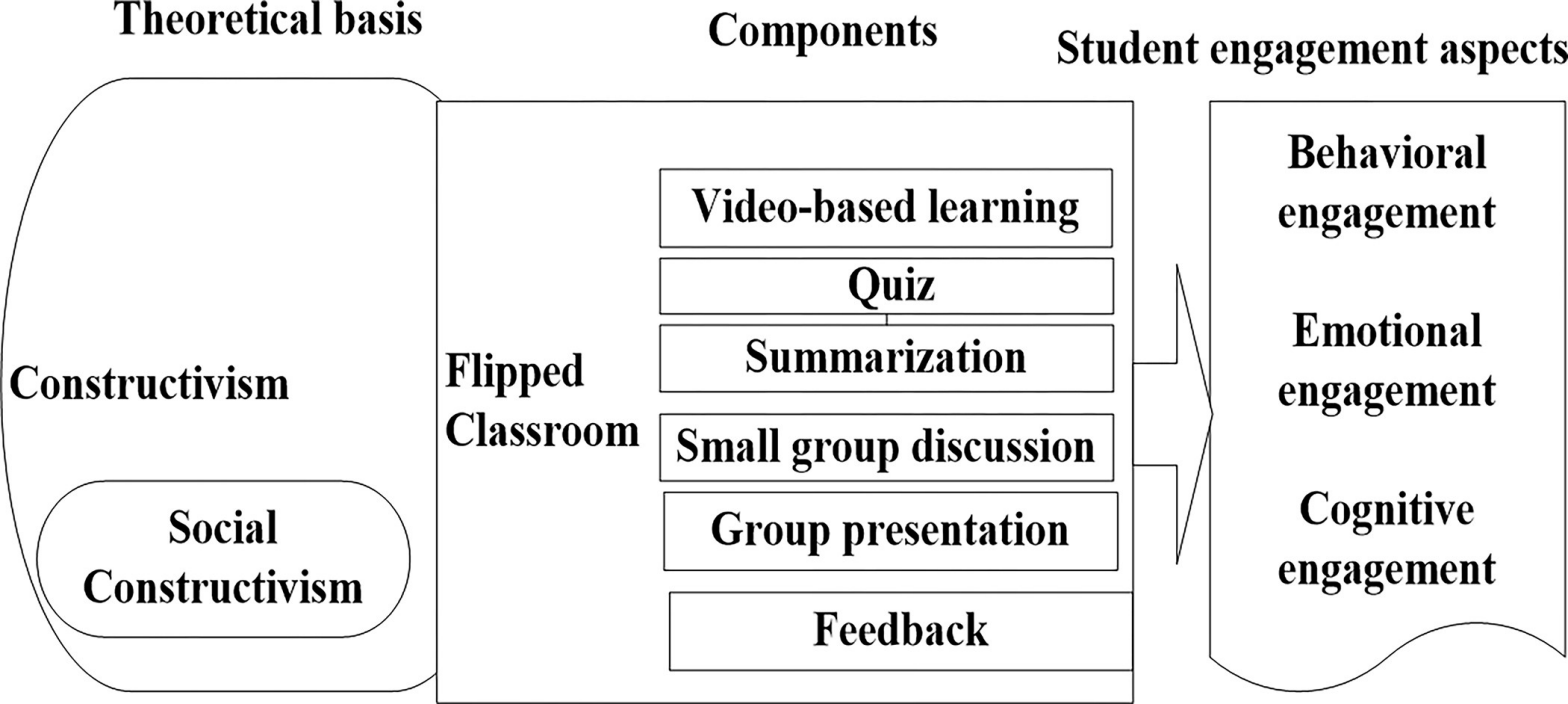
Study conceptual framework on student engagement using a flipped classroom at Mattu University, Ethiopia, 2023.

The current study employed the flipped classroom, guided by the approaches drawn from prior literature and considering the study institution settings. These included giving quick training to both teachers and students, creating incentive-filled video lectures, sharing the prepared materials ahead of time via accessible technology, summarizing the video lectures, conducting small group discussions, allowing group presentations, and offering feedback (Fig 1).

### Research questions

The purpose of the study was improving student engagement using flipped classroom instructional model. Specifically, the following basic question was raised and answered: to what extent does a flipped classroom enhance student engagement, including behavioral, emotional, and cognitive aspects?

## Methods and materials

Mixed-methods design was employed to achieve the purpose of this study, taking into consideration the advantages of the design over a quantitative and qualitative design by addressing weaknesses like lack of context, participant voices, and personal biases [34]. In particular, the study used the convergent design. The convergent parallel design is a type mixed-methods design that involves collecting and analyzing both quantitative and qualitative data, comparing results, and comparing them to determine if the findings support or contradict each other [35]. The current study employed the convergent parallel design to combine quantitative and qualitative components (QUAL+QUAN) with equal weights [34]. In general, the mixed-methods designis useful to integrate data from multiple sources for a thorough knowledge, flexibility in methodological interpretation, and triangulation of data for more reliable inferences and conclusions [36]. Furthermore, it helpful to ensures credibility, context, and diversity of views, ensuring the integrity of findings [37].

This study employed the mixed-methods designto strengthen the trustworthiness of the results, which incorporate different views from different sources. It was assumed that the two kinds of data gathered from both teachers and students could corroborate each other and indicateeffectively the extent to which a flipped classroom improves student engagement, including its three dimensions: behavioral, emotional, and cognitive.

### Research context and participants

The Ethiopian Higher Education Proclamation emphasizes that in order to provide a supportive learning environment for instructors and students alike, higher education institutions must provide enabling educational settings that promote student-centred strategies [38].

However, because most teachers employ a teacher-centered teaching strategy, a recent study discovered that the learning environment was not conducive to student engagement [39]. Furthermore, research revealed that a lack of experienced teachers was one issue that some Ethiopian universities encountered, which had negative impact on the quality of the teaching and learning processes [40]. Since Mattu University was believed to be experiencing comparable issues to other universities within the country, it was selected for this study. In response to those challenges, a flipped classroom teaching model was proposed, assuming it could bring about a significant change in the teaching style that depresses student engagement in the institution under investigation.

Two colleges were chosen for this study randomly, and from those colleges, two departments were picked at random. Two batches of departments were then chosen using the lottery method, one from each department. Accordingly, second- and fourth-year batches were identified from the "instructional technology" and "educational technology and information management" departments, respectively. Lastly, a lottery was used to select two of the major courses: "Teaching and Learning in a Digital Age" and "Basic Computer Programming." Along with their students, two teachers who were assigned to teach those courses became the participants in this study. It was agreed upon with those teachers to use both traditional teaching methods and the flipped classroom instructional style when teaching those courses. Accordingly, teachers used a traditional teaching approach for the first four weeks of those courses, as they usually did, and a flipped classroom approach for the last four weeks.

The total number of students registered for two courses was fifty-five (N = 55). Of those students, twenty (N = 20) were enrolled in educational technology and information management, while thirty-five (N = 35) were enrolled in information technology.

The flipped classroom instruction model was implemented between February 13, 2023, and April 2, 2023. The in-class learning sessions were 90 minutes each. The time allotted in advance guided major tasks, which were indicated in the conceptual framework, to be completed in class (Table 1).

**Table 1:** Instructional process for in-class learning.

Instructional activities	Time allotted
The teacher collects assignments.	5 minutes
Students take quizzes.	10 minutes
The teacher provides mini-lecture after quizzes.	10 minutes
The teacher provides motivating questions for discussion that align with pre-class foundational knowledge.	5 minutes
Students form groups with four or five individuals and have a discussion.	40 minutes
Groups present their views to the class.	15 minutes
The teacher offers feedback	5 minutes

### Instruments

This study involved both qualitative and quantitative data-gathering tools. Regarding the qualitative part, the researchers developed the guiding questions that were used to collect qualitative data through student focus groups (FGD) (S1 File), teacher interviews (S2 File), and observation methods (S3 File). Likert scales with values of "completely disagree," "disagree," "undecided," "agree," and "completely agree" were utilized to gather the quantitative data (S4 File). The instruments were used to collect data on the engagement levels of students who participated in the interventions.

Three categories—behavioral engagement, emotional engagement, and cognitive engagement—were used to arrange the tools.

The study’s instruments, particularly the Likert scale portion, underwent various review procedures. Firstly, all items were examined by different experts from different fields of study to check their validity. Seven academic staff members were then asked to review the items, representing a range of fields. Curriculum and instruction, psychology, educational technology, English language and literature, educational leadership, and educational research were among the disciplines represented by the experts in the field. The survey items were subjected to those experts for reviews and grading processes to examine the content validity of the instrument and tailor it to the study setting. On a 4-point grading scale, the panelist’s gave all items the following ratings: not relevant, slightly relevant, quite relevant, and highly important [41]. Scholars suggested that in order for an item to be retained and used, its content validity index (CVI) should always be more than 0.83 [40–42]. Accordingly, every item was examined got acceptance with computed content validity index (CVI) ranged from.86 to 1. However, in light of the expert comments, some of the items were reworded. Next, the pilot test was conducted with 356 participating students earlier at the university where the current study was conducted. The pilot test did not include the students who took part in the current study. Each item’s Cronbach’s coefficient was equal to or greater than 0.89. The Cronbach’s alpha (α) was calculated, and the coefficients ranged from 0.90 to 0.94 for the three aspect of student engagement, including behavioral, emotional, and cognitive engagement. Therefore, all items had no reliability problem, as all the Cronbach’s coefficients were above the minimal value of 0.7 [43].

### Data collection

The study involved both quantitative and qualitative data collection methods. The study used self-report surveys to collect the quantitative data, whereas classroom observation techniques, student focus groups, and teacher interviews were used to obtain the qualitative data.

Regarding quantitative data, by drawing comparisons between their experiences with traditional pedagogy and the flipped classroom teaching approach, fifty-five participant students provided the information that was required. The study collected quantitative data from the participant students in person before and after an intervention using Likert-scale questionnaires. Likert-scale surveys were used because they are easy to use, understandable, and most suitable for raters [44,45].The self-report survey used in this study was based on the assumption that the students who participated could readily communicate their impressions of what they had experienced in a flipped classroom.

Six students, two teachers, and two classroom observers participated in the qualitative data collection. Six students were chosen for the focus group discussions out of the fifty-five students, three from each discipline, in order to obtain qualitative data. The six participating students were selected by purposeful sampling in light of their enthusiastic participation in class.

The number six students was assumed to be sufficient because a focus group discussion could involve 5–10 participants [46]. In order to ascertain the shared perspectives and experiences of the student participants regarding the extent to which flipped classes enhanced engagement—including all of its dimensions—a focus group discussion was conducted.

An assistant moderator and one moderator were used for the data collection process. The assistant recorded the discussion using a cell phone, while the moderator led the discussion and took notes. It took an hour and a half to complete the discussion. All participants gave their approval for the discussion, including the recordings [47].

The semi-structured interviews were conducted with two teachers who participated in developing and applying the flipped classroom instructional model to their courses for four weeks. The two instructors were used as interviewees for two reasons: first, to strengthen the truthfulness of the study’s findings; and second, to collect data that could not be obtained from other sources. They included information about how well each student performed on assignments, assessments, and group projects that could be used as indicators for engagement. The interviews were facilitated by one moderator and one assistant moderator through field notes and mobile phone recordings based on the obtained consents [48]. The semi-structured interview was chosen due to its power, flexibility, and adaptability, allowing for in-depth information from informants [49].

Classroom observation is a widely used technique for collecting qualitative data related to educational research. It emphasizes comfort, rapport, and comprehension of important circumstances, allowing for real-time tracking and behavior analysis [50]. Specifically, the study employed structured classroom observation, focusing on specific indicators of student engagement and procedures for flipped classroom instructional model application [51].

As mentioned before in the "instrument" section, the observation check lists were examined by specialists prior to use in order to preserve authenticity and dependability [52]. Two experienced teachers, one from the study team and the other from the academic staff, made the observations in the classroom. To ensure that the triangulation was successful, we did not disclose the study’s expected outcomes to the observer, who was not a member of our research team [53]. In this study, classroom observations were required to gather first-hand information from classroom settings and supplement the data gathered from other sources.

In three rounds, the participants observed the classroom in the role of non-participants. The first round of observations was conducted before intervention during the first four weeks of regular classes, and the other two rounds took place during the second four weeks of intervention. To protect participants from disturbances that may remove them from actual experiences, a brief discussion was conducted with students, and necessary consent was obtained in advance. The observers recorded, captured photos, and conducted documentation based on the observation checklists [50]. Finally, both observers organized their reports independently based on the provided checklists and delivered the reports to the research team [51,52].Observers analyze physical setting, participants, roles, demographics, activities, conversation, and nonverbal communication to understand qualitative data, minimizing researcher influence and participant interactions for valuable insights [50].

### Data analysis

Focus group and interview audios that had already been recorded, along with discussion notes, was used to make transcriptions of the events. Notifying the participants of the data’s findings served as a means of member verification. The two observers provided their reports independently with images and videos. The data obtained from the two observers were transcribed and compared during analysis. Thematic data analysis was used to assess the qualitative data. The codes and quotations were utilized to analyze the data using the three dimensions of student engagement as themes [37,53,54].

The names of the participants were replaced with codes in order to maintain their confidentiality. IS1, IS2, and IS3 stood for the students selected from the information technology discipline, while ETS1, ETS2, and ETS3 stood for the students from the educational technology and information management disciplines. TF and TB codes were applied to the participating teachers. The two classroom observers were represented by the codes Cor1 and Cor2.

The quantitative data were analyzed using descriptive statistics, particularly percentiles and aggregated mean and paired sample t tests. While the descriptive analysis was employed to describe general background of the participants, paired sample t test, was utilized to compare the aggregated mean of pre-interventions and post interventions pertaining the opinions of the learners towards their engagements with flipped classroom [55]. Given that paired t-test computations could be performed with a sample size as small as the one used in this study, it was anticipated that fifty-five individuals would be adequate [56].

### Ethical consideration

The Jimma University, College of Education and Behavioral Science Institutional Review Board (IRB) provided a letter of ethics approval. The proposed research was examined by the institutional review boards (IRBs) to verify ethical behavior and that the requirements for exemption were met. After then, permission was requested from Mattu University, where the intervention was conducted, to deal with the selected disciplines. Following participant identification, conversations were held separately with the fifty-five students and the two respective course instructors. Clarifying the goal, research methodology, data collection techniques, and participant rights was a major focus of the talks to guarantee that participants made a free and informed choice to participate in the study. The study participants were guaranteed the freedom to discontinue participation at any time or to withdraw from the study altogether. Confidentiality was protected throughout the whole research process, it was assured. Ultimately, verbal consent was acquired from every participant via individual conversations [57]. Before any analysis was done, the transcripts of the focus group and interview discussions were delivered to the participants for verification. The intervention was executed between February 13, 2023, and April 2, 2023, following approval.

## Results of the study

The analysis focused on the extent to which the flipped classroom improved student engagement as compared to the students’ conventional class experiences. In this study, student engagement is defined and treated as having three elements: behavioral, emotional, and cognitive [8]. Regarding the demographic background of the participants, among the six students who provided qualitative data through focus group discussion, 50% were males and the remaining were females. Both of the teachers who took part in the interview were men. Among the participants in quantitative data, thirty-three males (or 60%) and twenty-two girls (or 40%) took part in interventions.

### Student behavioral engagement changes with flipped classroom model

As defined earlier, the student behavioral engagement stands for student participations in the lessons being taught in the classroom [58]. The summary table of codes, which provides the samples for codes and analysis themes, served as the foundation for the qualitative data analysis (Table 2). Student behavioral engagement is the first theme in the qualitative data analysis section. In relation to this theme, the student participants reported that the flipped classroom model could significantly increase their level of classroom engagement by enabling them to participate in all activities, turn in assignments on time, get ready for class, and the like. For example, discussant one (IS1) claimed that the new instructional model was able to increase his engagement in class activities to finish assignments on time, prepare for class material ahead of time, and actively participate in class discussions. Another participant (ETS1) shared his opinion that it would be better if other teachers also implemented the flipped classroom model. He described his experiences, saying that the model simplified classroom participation and the completion of assignments and quizzes for him through the lecture video that the lecturer provided in advance. Another participant stated that the flipped classroom approach was a better way to boost student participation than the teacher’s traditional method of instruction. She asserted that since she had already watched lecture videos, she possessed the basic knowledge necessary to fully participate in every class activity. She also mentioned that she successfully finished the quizzes that were offered with the support of the new approach (ETS 3).

**Table 2:** A Summary of table of codes.

Data Sources	Sample Codes	Themes
Student Focus Group Discussion	“Active participation in class,” complete given tasks better,” “get prepared for class better,” “ask questions actively,” etc.	Behavioral engagement
Teacher Interviews	“Better to fulfil academic roles in class,” “every student is able to complete assigned tasks,” “better to raise hands to respond,” etc.	
Classroom observations	“Better achievement to complete individual assignments,” “improved classroom activities,” “enhanced involvement to ask questions,” “improved attendance,” etc.	
Student Focus Group Discussion	“Improved interest in learning,” “better interactions among students,” “enhanced interactions between teacher and students,” “improved commitments,” etc.	Emotional engagement
Teacher Interviews	“Improved student interactions with one another,” “enhanced teacher-student communications,” “better confidence,” etc.	
Classroom observations	“Better interactions among students,” “friendly teacher-student interactions,”“improved self-confidence,” etc.	
Student Focus Group Discussion	“Improved understanding,” “better efforts to share knowledge,” “improved efforts to learn,” “better recall capacity,” etc.	Cognitive engagement
Teacher Interviews	“Improved assignment reports,” “improved recall,” ‘better efforts to learn,” “enhanced understanding,” etc.	
Classroom observations	“Better to grasp contents,” “batter to take note”; “intensive dialogue,” etc.	

Regarding the results of teacher interviews, similar to what their students had said, the participating teachers asserted that the benefits of the new model for increasing student behavioral engagement were paramount. One teacher (TB) indicated that the strategy worked well for helping students do their assignments, participate in class discussions, complete quizzes successfully, show up for class, attend class frequently, and meet other academic requirements. Another teacher also noted that the model assisted all students in fulfilling their academic obligations (TF).

Based on observations made in the classroom, one observer concluded that the model assisted all students in completing assignments on time, actively participating in class discussions, asking questions, successfully completing quizzes, presenting well, and attending class regularly (Cor1). The second observer reported the same evidence (Cors2).

The quantitative data collected showed that, with regard to the behavioral component, there was a substantial difference between conventional and flipped classrooms. The students assessed their behavioral engagement twice using the items that contained indicators such as “I complete individual assignments as directed by my teacher,” “I participate in group works with classmates,” “Whenever I have any questions during class, I ask my teacher,” “I study the given learning materials properly,” and “I participate in class discussions and answer questions.” Accordingly, the post-pre results showed that the observed change in their behavioral engagement was significant (t = 28.3, p < .001) (Table 3). This implies that the results of the two sets of data supported each other regarding the positive and significant effect that flipped classrooms had on student behavioral engagement.

**Table 3:** The improvement observed with a flipped classroom in student behavioral engagement.

		Paired Differences					t	df	Sig. (2-tailed)
		Mean	Std. Deviation	Std. Error Mean	95% Confidence Interval of the Difference				
					Lower	Upper			
Pair 1	Post-pre intervention	9.18	2.4	.324	8.53	9.83	28.3	54	.000

### Student emotional engagement changes with flipped classroom model

This represents the way a student’s feelings respond to their academic work, instructors, and peers in order to encourage a dedication to learning [8]. The qualitative data results demonstrated that students’ emotional engagement with the flipped classroom was much higher than in the conventional classroom. In focus group discussions, one of the student participants highlighted the enhancements in peer, student-teacher, and learning resource interaction that came about as an outcome of the flipped classroom model (IS4). According to him, the approach “would foster close relationships among students, between student and teacher, as well as between students and lessons.” Another participant asserted that the model supported his desire and dedication to establishing positive connections with his teacher and peers more than the traditional method did (IS1). The remaining participants also stated that, in terms of fostering emotional engagement, the new model was superior to the traditional one.

Teachers also asserted that the flipped classroom model was more effective in fostering their emotional engagement compared to their conventional strategy. One teacher, for example, reported that the model was more effective at fostering positive learning environments because it improved student-teacher interaction and sparked students’ enthusiasm and dedication to carrying out their instructional duties (TF). Another instructor who has used flipped learning reported that the model significantly increased students’ emotional engagement by encouraging curiosity, confidence, and a dedication to supporting one another (TB).

The classroom observers also reported that the flipped class was a superior method for addressing the emotional side of engagement. The first observer (Cor1) noted that he witnessed a significant desire on the part of students to work together, as well as more confidence in asking questions and expressing ideas to both the teacher and peers. Another observer (Cor2) noticed that the students’ concentration and commitment to acting out their roles were better than what he had seen in previous conventional classes (Cor1).

Similar to other aspects of engagement, the students assessed their emotional engagement twice using the provided indicators. The items contained indicators such as “I have confidence that I can do well in the class.”; “My classroom is an interesting place to be.”; “I am energized by the activities we undertake in the classroom.”; “I find the course materials fascinating; and “I enjoy working on tasks with my classmates.”. According to student reports, the computed findings demonstrated significant improvements in emotional engagement with a flipped classroom (t = 38.86, p < .001) (Table 4). This implies that the results of the two sets of data supported each other regarding the positive and significant effect that flipped classrooms had on student behavioral engagement.

**Table 4:** The improvement observed with a flipped classroom in student emotional engagement.

		Paired Differences					t	df	Sig. (2-tailed)
		Mean	Std. Deviation	Std. Error Mean	95% Confidence Interval of the Difference				
					Lower	Upper			
Pair 1	Post-pre Intervention	10.42	2.0	.27	9.9	10.95	38.86	54	.000

### Student cognitive engagement changes with flipped classroom model

Cognitive engagement involves understanding complex ideas through reading, task completion, self-control, concentration, and metacognitive techniques, which require effort, persistence, and in-depth thought, all linked to learning [4,58,59].

As described next, the qualitative data demonstrated that the flipped classroom model significantly and positively improved student cognitive engagement. For instance, in a focus group, one participant (IS2) stated that he experienced the model as an effective method for improving one’s attention in class, understanding the provided learning materials, and completing the assigned tasks with better quality. Comparing it to his prior engagement experiences in traditional classrooms, another discussant (ETS3) noted that the new strategy allowed him to finish individual tasks with greater quality and understand effectively the portions covered with the new instructional approach. Every participant in the focus group discussions noticed the same indication of the notable improvement in the cognitive component of student engagement.

Similarly, it was noted during the teachers’ interview that the new model exceeded their conventional strategy for improving the quality of the students’ work and comprehension. For instance, teacher TF asserted that the flipped classroom effectively enhanced the cognitive aspect of student engagement by supporting the students in completing assigned tasks with better quality, participating in class with better understanding, sharing one’s understanding with peers, making group presentations with better understanding, and other similar qualities. The instructor participant, TB, also reported that he saw improvements in the students’ cognitive abilities as evidenced by their greater effort to complete assignments, improved concentration in class, completion of projects with minimal mistakes possible, and other such behaviors.

Furthermore, reports from classroom observations demonstrated that the model could enhance student cognitive engagement. This was proved by achieving the teacher’s standards for group presentations, recalling what they studied, taking notes in class, and having a better grasp of the course materials (Cor1). The second observer noticed positive changes as each student made an effort to finish their assignments, including sharing understandings in class, paying attention in class, trying diligently to fulfil roles, completing assignments in line with teacher expectations, and performing similar tasks (Cor2).

Regarding the quantitative data, the study used some indicative items to measure student cognitive engagement. The items contained statements such as “Despite how challenging the lessons are, I continue to try them.”; “I frequently try to comprehend things better."; “I understand what I’m doing."; “I try to put new concepts I’ve learned into my own words."; and “I usually review and correct my assignments before I submit.”. As shown in Table 5, the computed post-pretest findings demonstrated that the flipped classroom could significantly enhance student cognitive engagement (t = 35.17, p < .001).This suggests that the two data sets’ results complemented one another’s findings about the significant and substantial impact that flipped classes had on students’ cognitive engagement (Table 5).

**Table 5:** The improvement observed with a flipped classroom in student cognitive engagement.

		Paired Differences					t	df	Sig. (2-tailed)
		Mean	Std. Deviation	Std. Error Mean	95% Confidence Interval of the Difference				
					Lower	Upper			
Pair 1	Post-pre Intervention	10.1	2.13	.29	9.5	10.7	35.17	54	.000

### Converging the results of the two sets of data

This section presents a comparison of the results from the two data sets in order to provide clarity on the conclusions drawn. As mentioned earlier, the current study used a flipped classroom instruction model to increase student engagement in all three of its components: behavioral, emotional, and cognitive. Within the context of this study, behavioral engagement refers to a student’s involvement in the activities associated with the lessons being taught in the classroom, while emotional engagement is a student’s feelings towards their academic work, teachers, and classmates. Cognitive engagement involves understanding complex ideas through reading, task completion, self-control, concentration, and metacognitive techniques, requiring effort, persistence, and in-depth thought, all linked to learning [8].

The findings from both data sets concurred with one another regarding the impact of a flipped classroom instruction approach on student behavioral engagement. By enabling on-time assignment completion, pre-class preparation, active participation in class discussions, asking questions, studying materials, and other similar activities, the flipped classroom approach boosted student behavioral engagement, according to the qualitative data analysis. The students examined the behavioral aspect of their engagement using self-report survey. And the calculated data revealed that the observed change was significant (t = 28.3, p < .001), indicating improved engagement. This implies that the results of the two data sets support each other.

In addition, the flipped classroom model significantly enhances emotional student engagement, fostering positive relationships with teachers, classmates, and learning resources, boosting enthusiasm, confidence, collaboration, and dedication, as confirmed by qualitative data. The results of the quantitative results showed similar results to those of the qualitative ones. The calculated results showed significant and positive changes in student emotional engagement due to a flipped classroom (t = 38.86, p < .001).

Furthermore, a new learning model dramatically improved students’ cognitive engagement, exceeding traditional approaches in terms of making consistent efforts to master lessons, understanding learning materials, task quality, and the like, according to the results of the qualitative data analysis. Like other engagement dimensions, the quantitative data results complement the qualitative findings about the impact of the flipped classroom on students’ cognitive engagement. The computed findings demonstrated that a flipped classroom had a significant positive impact on students’ cognitive engagement (t = 35.17, p < .001).

## Discussions

The emergence of novel instructional technology, such as the flipped classroom instruction model, has brought about a revolutionary shift in the way that instruction is conducted [60]. By combining traditional lecture-based methods with online video lectures and problem-solving in class, the flipped classroom model gives students more control over the learning process and allows them to actively generate meaning. This method encourages students to actively explore their subject [29]. The flipped classroom design, which allows students to study course content at home with instructor support, has been found to be more effective in improving student engagement than face-to-face education [61]. As a result of this, the flipped classroom model, among many advantages, can increase student learning motivation and boost student performance, which may lead to positive attitudes [62]. In addition, the flipped classroom model enhances student engagement by creating a more pleasant learning environment and promoting key learning factors like peer commitment, recognition, safety, and teacher-student relationships, as revealed in previous studies [63]. Consistent with the prior investigations, the combined outcomes of qualitative and quantitative data sets demonstrated that the flipped classroom model outperformed the conventional teaching approach in raising student engagement in behavioral, emotional, and cognitive domains, as revealed in the present study. Moreover, a flipped classroom model enhances student engagement by assisting in meeting for assignments, motivating them, and promoting deep learning, as confirmed by research by Huang et al., 2019 [64]. The flipped classroom model, according to a study by Ayçiçek & Yelken (2018), can significantly improve student engagement in classes by enabling independent study and interaction with teachers and peers [65]. In the present study, consistent with those findings, the new instruction model increased student behavioral engagement by supporting each person in completing tasks on time, preparing ahead of time for class, participating fully in class discussions, and engaging in related activities. Additionally, this method helped students build strong bonds with instructors, peers, and educational materials, which increases enthusiasm, confidence, teamwork, and commitment. The model also brought about a considerable positive influence on students’ cognitive engagement, overtaking traditional techniques in terms of mastering courses, understanding materials, task quality, and the like.

This study had limitations. The sample size was a factor in the first limitation. In the current study, only two teachers, fifty-five students, and two courses from two distinct fields were involved. An additional limitation was related to the time frame. The flipped classroom model, which was novel in study environments, was implemented in a short amount of time. There may have been a recall bias among the student participants due to the short duration. Given these limitations, it is unrealistic to recommend that the findings of this study be directly applicable to different disciplines or institutions as a whole. Future research may deal with the effects of the flipped classroom model on student engagement, considering other education levels. Using a longitudinal study design, future research may examine the impact of the flipped classroom instruction model on academic achievement, learning motivation, academic self-efficacy, and related topics in Ethiopian educational settings.

## Conclusions

The study demonstrated how the flipped classroom approach greatly improves student involvement in every way. The students in flipped classrooms were shown to be especially committed to actively engaging in the learning process. They responded positively and promisingly to their teachers, peers, and lessons. They have shown exceptionally high levels of effort in trying to understand the material. The findings are in line with earlier research from various foreign higher education institutions, yet there aren’t many studies on flipped classes done in Ethiopian environments. The proposed framework for designing and implementing flipped classroom model was found effective in the study contexts. Thus, the study has approved that the flipped classroom is a promising innovative method that can enhance student engagement, which may be a crucial factor for high-quality learning. However, extended and thorough investigations are appropriate for the future, considering the different education levels and institutional settings in Ethiopia.

## Supporting information

S1 File Student FGD guiding question. (DOCX)

S2 File Teachers interview guiding questions. (DOCX)

S3 File Classroom observation checklists. (DOCX)

S4 File Student self-report survey questionnaires. (DOCX)
