# Supplementary material for: Improving student engagement with a flipped classroom instruction model in Ethiopian higher education institutions: The case of Mattu University
Source: PLoS One. 2024 Oct 2;19(10):e0307382. doi: 10.1371/journal.pone.0307382 (PMC11446460; doi:10.1371/journal.pone.0307382)
Supplement: S1 File — (DOCX) [file pone.0307382.s001.docx]

**Mattu University**

**Students focus group discussion (FGD guiding questions**

***Introduction***

As you remember from the previous eight weeks, your teacher used two distinct teaching modalities to deliver the lesson. For the first four weeks of the session, the teacher taught it using the traditional method. He employed the new teaching strategy for the final four weeks of the course. In order to complete your responsibilities as a student, you had to participate in both strategies. This focus group discussion aims to compare how engaged you are in fulfilling your duties using the two different teaching techniques. Therefore, we kindly request that you compare the two methods—the old and the new one—in order to assess the level of engagement that you and your colleagues were achieving.

Your private and confidential information will not be used in any written or public data analyses resulting from the study. Please be aware that this is entirely voluntary for you to participate in and that you can stop at any time without facing any consequences. As volunteers, you will not receive any immediate rewards, but your participation is crucial to the success of the study. As a result, we would like to hear your honest opinions on your experiences attending the flipped classroom lessons.

**Guiding question**

1. In what ways do the two modes of student engagement differ when it comes to fulfilling the assigned tasks?

- To complete the tasks on time
- Participate in group discussions
- ,To ask questions
- To attend class regularly
- To put on a strong act of working in class.
- To achieve good results in tests, etc.

1. Regarding the response to your friend, teacher, and course contents, how do you see your own feelings in both modalities and those of each student? When presenting your ideas, keep the following points in mind:

- Student interactions with peers
- Student interaction with teacher
- Interest to study the lesson
- Confidence to play one’s roles,
- commitment, etc.

1. What opinions do you have about how well the two instructional strategies foster student understanding? Compare and contrast the two approaches. To compare the two approaches, take into account the following points:

- Assignments quality
- Taking notes using one’s words
- Recalling what you learned
- Sharing with peers what you gained
- Persistent efforts to master the lesson,
- Relating the lessons to the real world,
- Attention in class, etc.
