# Supplementary material for: Improving student engagement with a flipped classroom instruction model in Ethiopian higher education institutions: The case of Mattu University
Source: PLoS One. 2024 Oct 2;19(10):e0307382. doi: 10.1371/journal.pone.0307382 (PMC11446460; doi:10.1371/journal.pone.0307382)
Supplement: S2 File — (DOCX) [file pone.0307382.s002.docx]

**Mattu University**

**Teachers interview guiding questions regarding the effects of a flipped classroom on student engagement**

**Introduction**

You remember that you were teaching your course using two different teaching approaches: conventional or using your usual methods and a flipped classroom instruction model for the last eight weeks. You taught your course for the first four weeks using the conventional one, whereas the last four weeks used the flipped classroom model. We respectfully ask for your opinion on the differences between the traditional and flipped classes in terms of student engagement.

We appreciate your commitment to sharing your insightful ideas with us in advance.

1. In comparison to the conventional model, what differences did you notice with the flipped classroom regarding student involvement in learning activities? When you compared the two instructional approaches, what gains in student performance did you see in terms of finishing the assigned work and engaging in class activities?
2. Did you notice that the new strategy helped your students participate actively in group discussions, unlike the old method?
3. To what extent did the flipped classroom encourage students to communicate with you and ask for support from you, as compared to the usual approach?
4. What did you notice about your students' confidence in their ability to participate in class discussions, give presentations, encourage one another, and answer your questions?
5. When comparing the flipped classroom to the conventional method, what improvement did you see in your students' motivation to do their assignments in class?
6. In contrast to the conventional approach, what did you notice about the effect of a flipped classroom on student interactions with one another in class?
7. What did you observe regarding your students' efforts in completing challenging questions in the flipped classroom compared to the traditional one?
8. To what extent did the new teaching approach improve your students understanding of the lesson?
9. To what extent did the new approach encourage your students to share the knowledge they gain with their peers?
