# Supplementary material for: Improving student engagement with a flipped classroom instruction model in Ethiopian higher education institutions: The case of Mattu University
Source: PLoS One. 2024 Oct 2;19(10):e0307382. doi: 10.1371/journal.pone.0307382 (PMC11446460; doi:10.1371/journal.pone.0307382)
Supplement: S3 File — (DOCX) [file pone.0307382.s003.docx]

**Mattu University**

**Classroom Observations Checklists for Flipped Classroom Implementation**

Round_________

Date: _______

Department:_______________________________

Course Title: ______________________________Course Code_____________

Lesson Title:______________________________________

Number of enrolled students: Male_____ Female ______Total______

Student available at class: male____ Female ______Total_____

Total number of groups in class________ Number of students in each group:____

***Guiding questions***

To what extent do students come to class with completed individual assignments?

__________________________________________________________________________________________________________________________________________________________________________________________________________________________________________

To what extent do the students participate in class discussions?

__________________________________________________________________________________________________________________________________________________________________________________________________________________________________________

To what extent do students actively ask questions in class?

__________________________________________________________________________________________________________________________________________________________________________________________________________________________________________

To what extent do students attend class regularly?

__________________________________________________________________________________________________________________________________________________________________________________________________________________________________________

To what extent do students feel free to communicate with their teacher and peers?

_______________________________________________________________________________________________________________________________________________________________________________________________________________________________________

How much passion do students show in participating in class activities?

_______________________________________________________________________________________________________________________________________________________________________________________________________________________________________

How is the student’s confidence in participating in class activities?

________________________________________________________________________________________________________________________________________________________________________________________________________________________________________

How is the student's understanding while working on the tasks given by the teacher?

____________________________________________________________________________________________________________________________________________________________

To what extent do students complete the given tasks up to the teacher’s standard?

____________________________________________________________________________________________________________________________________________________________

To what degree does each student try to impart the knowledge they learn on their peers?

____________________________________________________________________________________________________________________________________________________________

How focused are the pupils during class?

____________________________________________________________________________________________________________________________________________________________

How much do students try to take notes using their own words?

________________________________________________________________________________________________________________________________________________________________________________________________________________________________________

Name and sign of the classroom observer: ___________________________________________
