# Supplementary material for: Improving student engagement with a flipped classroom instruction model in Ethiopian higher education institutions: The case of Mattu University
Source: PLoS One. 2024 Oct 2;19(10):e0307382. doi: 10.1371/journal.pone.0307382 (PMC11446460; doi:10.1371/journal.pone.0307382)
Supplement: S4 File — (DOCX) [file pone.0307382.s004.docx]

**Mattu University**

**Questionnaires to be filled out by students** **on the effects of flipped classroom on student engagement**

**Introduction**

These questionnaires are used to collect data for the study, which aims at examining the effects of a flipped classroom instruction model on student engagement in academic matters. The accuracy of the information is determined by your sincere responses. As a result, we respectfully ask that you provide accurate information. The information you give will be kept private, and the information gathered will only be used for this study, the researcher guarantees.

**Part one: General information**

College____________________________Department________________________ Batch or year___________

Sex:

Male

Female

*Note*

- No need of writing your name√
- Please mark each choice with a "√" to reflect the degree of your engagement based on the provided items.

We appreciate your cooperation in advance.

**Part two: Indicators to assess your degree of engagement**

*1= I strongly disagree, 2= I Disagree, 3= I can’t decide, 4= I agree, 5= I strongly agree*

| SN | Item | 1 | 2 | 3 | 4 | 5 |
| --- | --- | --- | --- | --- | --- | --- |
|  | **Behavioral engagement** |  |  |  |  |  |
| 1 | I complete an individual assignments timely |  |  |  |  |  |
| 2 | I actively participate in class discussions. |  |  |  |  |  |
| 3 | I actively participate in group works with classmates |  |  |  |  |  |
| 4 | Whenever I have any questions during class, I ask my teacher. |  |  |  |  |  |
| 5 | I regularly attend class |  |  |  |  |  |
| 6 | I tried really hard to work in class. |  |  |  |  |  |
| 7 | I score a good grade with quizzes. |  |  |  |  |  |
| 8 | I carefully watched the provided video lectures prior to class. |  |  |  |  |  |
|  | **Emotional engagement** |  |  |  |  |  |
| 1 | My teachers support me whenever I need it. |  |  |  |  |  |
| 2 | I consider myself to be a vital component of my learning team. |  |  |  |  |  |
| 3 | I am energized by the activities we undertake in the classroom. |  |  |  |  |  |
| 4 | My classroom is an interesting place to be. |  |  |  |  |  |
| 5 | I usually feel happy about the activities that I experience in courses. |  |  |  |  |  |
| 6 | I often feel that I am learning valuable things in class. |  |  |  |  |  |
| 7 | I find the course materials fascinating. |  |  |  |  |  |
| 8 | I enjoy working on projects or tasks with my classmates. |  |  |  |  |  |
| 9 | I am friendly with my instructor to get academic support. |  |  |  |  |  |
| 10 | I have confidence that I can do well in the class. |  |  |  |  |  |
|  | **Cognitive engagement** |  |  |  |  |  |
| 1 | Despite how challenging the lessons are, I continue to try. |  |  |  |  |  |
| 2 | I share what I have learned in class with my peers. |  |  |  |  |  |
| 3 | I frequently try to comprehend things better. |  |  |  |  |  |
| 4 | I understand what I’m doing. |  |  |  |  |  |
| 5 | I recall important course content after class. |  |  |  |  |  |
| 6 | I attend class with my best attention. |  |  |  |  |  |
| 7 | I try to put new concepts I've learned into my own words. |  |  |  |  |  |
| 8 | I usually review and correct my assignments before submission |  |  |  |  |  |
